# Ultrastructural and Molecular Analyses Reveal Enhanced Nucleolar Activity in *Medicago truncatula* Cells Overexpressing the *MtTdp2α* Gene

**DOI:** 10.3389/fpls.2018.00596

**Published:** 2018-05-11

**Authors:** Anca Macovei, Matteo Faè, Marco Biggiogera, Susana de Sousa Araújo, Daniela Carbonera, Alma Balestrazzi

**Affiliations:** ^1^Department of Biology and Biotechnology ‘L. Spallanzani’, Pavia, Italy; ^2^Instituto de Technologia Quìmica e Biologica António Xavier, Universidade Nova de Lisboa, Lisbon, Portugal

**Keywords:** etoposide, *Medicago truncatula*, nucleolus, transmission electron microscopy, tyrosyl-DNA phosphodiesterase

## Abstract

The role of tyrosyl-DNA phosphodiesterase 2 (Tdp2) involved in the repair of 5′-end-blocking DNA lesions is still poorly explored in plants. To gain novel insights, *Medicago truncatula* suspension cultures overexpressing the *MtTdp2α* gene (Tdp2α-13C and Tdp2α-28 lines, respectively) and a control (CTRL) line carrying the empty vector were investigated. Transmission electron microscopy (TEM) revealed enlarged nucleoli (up to 44% expansion of the area, compared to CTRL), the presence of nucleolar vacuoles, increased frequency of multinucleolate cells (up to 4.3-fold compared to CTRL) and reduced number of ring-shaped nucleoli in Tdp2α-13C and Tdp2α-28 lines. Ultrastructural data suggesting for enhanced nucleolar activity in *MtTdp2α*-overexpressing lines were integrated with results from bromouridine incorporation. The latter revealed an increase of labeled transcripts in both Tdp2α-13C and Tdp2α-28 cells, within the nucleolus and in the extra-nucleolar region. *MtTdp2α*-overexpressing cells showed tolerance to etoposide, a selective inhibitor of DNA topoisomerase II, as evidenced by DNA diffusion assay. TEM analysis revealed etoposide-induced rearrangements within the nucleolus, resembling the nucleolar caps observed in animal cells under transcription impairment. Based on these findings it is evident that *MtTdp2α*-overexpression enhances nucleolar activity in plant cells.

## Introduction

The nucleolus is a subnuclear entity with dynamic structure, where ribosomal RNA (rRNA) is synthesized and processed (Cisterna and Biggiogera, 2010; Shaw and Brown, 2012; Stepinski, 2014; Dvorackova et al., 2017). This self-organizing structure, modulated by endogenous and exogenous signals, is involved in stress sensing through the ‘nucleolar surveillance systems’ so far deeply investigated in animals (Tsai and Pederson, 2014; Lam and Trinkle-Mulcahy, 2015). Stimuli that target ribosome biogenesis result in nucleolar stress and trigger either p53-dependent or independent pathways to maintain cell homeostasis, as recently reviewed by Russo and Russo (2017). In the p53-dependent signaling pathways involving the MDM2 (mouse double minute) oncoprotein, the ribosomal proteins rpL5 and rpL11 control p53 levels by interacting with 5S rRNA within the 5S RNP (ribonucleoprotein particle) trimeric ribosomal subcomplex (Sloan et al., 2013). The p53-independent responses involve the ribosomal protein rpL3 which, under nucleolar stress, participates in the modulation of DNA repair by inhibiting the non-homologous end joining (NHEJ) repair pathway (Esposito et al., 2014).

The study of the crosstalk between the nucleolus and the DNA damage response (DDR) has been recently recognized as a challenging issue (Ogawa and Baserga, 2017). To date, nucleolar proteomics has revealed an increasing number of DNA repair components localized within the human nucleolus, among which is the DNA glycosylase APEX1 (Apurinic/apyrimidinic endodeoxyribonuclease 1) required for accurate and efficient synthesis of ribosomes, besides its function in the BER (base excision repair) pathway (Vascotto et al., 2009). A dual role in DNA repair and ribosome biogenesis has been suggested for the Werner syndrome RecQ like helicase (WRN) (Shiratori et al., 2002) and the Bloom syndrome RecQ like helicase (BLM) (Tikoo et al., 2013). In the nucleolus, the direct interaction of BLM helicase with DNA topoisomerase (topo) I, allows their coordinated action in the control of DNA/RNA hybrids production and relaxation of supercoiled DNA during transcription (Grierson et al., 2013).

Tyrosyl-DNA phosphodiesterases (Tdps) are multifaceted DNA repair enzymes able to remove the 5′- and 3′-end blocking DNA lesions, among which are topo/DNA covalent adducts (Pommier et al., 2014). Tdps represent interesting targets for the study of the nucleolar surveillance systems as well as the cross-talk between the nucleolus and DDR (Vilotti et al., 2011, 2012; Donà et al., 2013). Topological constrains accumulated within the DNA molecule are solved by DNA topoisomerases through the transient breakage of single or double DNA strands. During the catalytic cycle, the enzyme becomes covalently linked to DNA through a phosphotyrosyl bond. The topo/DNA covalent complexes are usually resolved, unless they are formed in the close proximity of damaged DNA sites or in the presence of specific inhibitors. In this case, they are converted into cytotoxic double strand breaks (DSBs) (Vos et al., 2011). Tdp1 and Tdp2 enzymes were initially classified in animal cells based on their ability to process 3′- and 5′-phosphotyrosyl covalent bonds of irreversible topo I- and topo II-DNA complexes (Pommier et al., 2014). However, the findings that they can interact with several key DDR players, e.g., PARP1 [poly (ADP-ribose) polymerase 1], XPF-ERCC1 (xeroderma pigmentosum group F-excision repair cross-complementation group 1), MRE11 (meiotic recombination 11) and Ku, have provided evidence of novel cross-talk routes between repair pathways (Pommier et al., 2014).

In plants, the stress-dependent reorganization of the nucleolar structure and the presence of a nucleolar checkpoint that activates programmed cell death (PCD) in relation to the Tdp1 function has been previously suggested by Donà et al. (2013), based on investigations carried out in the model legume *Medicago truncatula*. Differently from animals, a small *Tdp1* gene family is found in the plant kingdom, composed of *Tdp1α* and *Tdp1β* genes encoding distinct Tdp1 isoforms (Macovei et al., 2010). Transgenic *M. truncatula* plants with post-transcriptional silencing of the *MtTdp1α* gene showed impaired ribosome biogenesis, as revealed by RNA-Seq. This finding highlighted the essential role of Tdp1α in the nucleolar compartment (Donà et al., 2013). Accordingly, the collapsed nucleolar architecture observed in the *MtTdp1α*-depleted cells was consistent with a defect in rDNA transcription. Interestingly, RNA-Seq analysis of *MtTdp1α*-depleted plants revealed the differential regulation of genes encoding the ribosomal proteins BOP1 (Block of Proliferation 1), rpL23 and rpL10 (Donà et al., 2013) that participate in the modulation of ribogenesis under stress conditions, in both animal and plant cells. Indeed, the PeBoW (Pescadillo, BOP1, and WDR12) complex as well as the GTP-binding protein nucleostemin, have been recently demonstrated to play a key role in the stress-mediated control of ribosome biogenesis in *Arabidopsis* (Jeon et al., 2015; Ahn et al., 2016). To date, the NAC transcription factor ANAC082 is the only known component with a clarified role as a mediator of the nucleolar pathway in plants (Ohbayashi et al., 2017).

The human Tdp2 enzyme (EC number 3.1.4.), also known as TTRAP (TRAF and TNF receptor-associated protein), is not only required for repair of topo II-induced DSBs (Cortes Ledesma et al., 2009; Zeng et al., 2011) but also participates in signaling pathways which regulate cell proliferation/differentiation and apoptosis (Li et al., 2011; Varady et al., 2011). The lack of Tdp2 impairs ribosome biogenesis in animal cells and it has been hypothesized that the protein might be involved in the cross-talk between cytoplasm and nucleolus under stress conditions (Vilotti et al., 2012). In *M. truncatula* plants, the overexpression of *MtTdp2α* gene, encoding the α isoform of Tdp2 enzyme, is associated with enhanced DNA repair ability and tolerance to osmotic and heavy metal stress (Confalonieri et al., 2014; Faè et al., 2014). In a recent study, Araujo et al. (2016) demonstrated that *MtTdp2α* gene overexpression in *M. truncatula* cell suspension cultures resulted in increased cell viability and an extended exponential growth phase. Besides, the *MtTdp2α*-overexpressing suspension cultures revealed higher expression levels of antioxidant genes as well as the up-regulation of DDR genes (Araujo et al., 2016). Based on these premises, the aim of the present work was to study the role played by Tdp2 in the plant nucleolus using *MtTdp2α*-overexpressing *M. truncatula* cell suspension cultures. The nucleolar ultrastructural morphology and transcription rates, as well as the response to etoposide, an interfacial topo II poison (Wu et al., 2011; Montecucco et al., 2015), were investigated.

## Materials and Methods

### Cell Cultures and Treatments

The *M. truncatula* Gaertn. (M9-10a genotype) cell suspension cultures used in the present work were derived from the *MtTdp2α*-overexpressing plant lines Tdp2α-13c and Tdp2α-28 previously obtained by Confalonieri et al. (2014) as follows. The binary vector pART-Tdp2α containing the 35SCaMV-Tdp2α-OCS cassette was transferred by electroporation into EHA105 *Agrobacterium tumefaciens* strain and the resulting engineered strain EHA105-pTdp2α was used to transform the *M. truncatula* leaf explants. *A. tumefaciens*-mediated transformation of M9-10a leaf explants and *in vitro* regeneration were performed according to Confalonieri et al. (2009). Regenerated Tdp2α and control transgenic lines were maintained *in vitro* on a modified (10 g l^-1^ sucrose) MS030A medium and propagated using stem nodal explants. Plantlets were maintained in a climate chamber at 22–24°C with a 16-h light/8-h dark cycle photoperiod and a photosynthetic photon flux of 65–70 μMol m^-2^ s^-1^ under a cool white fluorescence lamp. *M. truncatula* Gaertn. (M9-10a genotype) cell suspension cultures were subsequently obtained as described by Araujo et al. (2016). Leaf explants, excised from *in vitro* grown plants of the *MtTdp2α*-overexpressing plant lines (Tdp2α-13c and Tdp2α-28) and control (CTRL) line (Confalonieri et al., 2014) were transferred to B5 medium (Gamborg et al., 1968) supplemented with 2,4-dichlorophenoxyacetic acid (2,4-D, 0.5 mg l^-1^, Duchefa Biochemie, Harleem, Netherlands) and maintained at 27°C in the dark for 2 weeks. The resulting calli were excised from explants and transferred to liquid MS medium (Murashige and Skoog, 1962) containing 2,4-D (0.25 mg l^-1^) and kinetin (0.25 mg l^-1^, Duchefa Biochemie). *M. truncatula* suspension cultures were routinely propagated in the same medium. Cells were subcultured every 8 days by transferring 5–45 ml of fresh medium and incubated at 80 rpm at 27°C in the dark. Treatments with etoposide (75, 150, and 300 μM; Sigma-Aldrich) were carried out as described by Locato et al. (2006). The topo II inhibitor was added to 4-day-old proliferating cell suspension cultures. Aliquots were then collected at the indicated time point (6 h) for molecular analyses and stored in liquid N_2_.

### Transmission Electron Microscopy (TEM)

Aliquots (2–3 ml) of 4-day-old *M. truncatula* cell suspension cultures (CTRL and *MtTdp2α*-overexpressing lines) were collected and fixed with 1.5% glutaraldehyde (Sigma-Aldrich) for 3 h at room temperature. Cells were rinsed in phosphate-buffered saline (PBS, pH 7.2) overnight, post-fixed in 1% aqueous OsO_4_ (Sigma-Aldrich) and embedded in epoxy resin. Sections were stained with uranyl acetate and lead citrate (Sigma-Aldrich) and observed with a Zeiss EM900 electron microscope equipped with a 30 mm objective aperture and operating at 80 kV.

### Morphometric Analysis

For morphometric analysis, all specimens were observed with a Zeiss EM900 electron microscope equipped with a 30 mm objective aperture and operating at 80 kV. Images were acquired at a fixed magnification (20,000×) and submitted to morphometric analyses using the software ImageJ. The surface area of 30 nucleoli per sample was measured. On the same samples, parameters such as the number of nucleolar vacuoles per nucleolus, number of nucleoli per cell, number of multinucleolate cells, and number of ring-shaped nucleoli, were determined. The results are expressed as mean values ± SD.

### Bromo-uridine Incorporation

Bromo-uridine (BrU) (Sigma-Aldrich) was dissolved in the culture medium and added to 4-day-old *M. truncatula* cell suspension cultures at the final concentration of 100 mM. Incubation was carried out for 1 h. Cells were then rapidly rinsed in fresh medium and processed for TEM as described by Trentani et al. (2003). An anti-BrU monoclonal antibody (rat anti-BrdU, Sera Lab, Hayworth Heats, United Kingdom) (Jaunin et al., 1998) was used at 1:5 dilution, corresponding to 5 mg ml^-1^. After rinsing with PBS containing 0.1% Tween and PBS, the grids were incubated with the specific secondary antibodies coupled with colloidal gold (1:20 in PBS) for 1 h at room temperature. Sections were finally stained with terbium citrate (Sigma Aldrich) 0.2 M pH 8.5 for 30 min at room temperature to detect RNA (Biggiogera and Fakan, 1998). The cells were observed at Zeiss EM900 electron microscope equipped with a 30 mm objective aperture and operating at 80 kV.

### DNA Diffusion Assay

DNA diffusion assay was performed as originally reported by Singh (2003) with the modifications recently described for plant cells by Macovei et al. (2017). Aliquots (300 μl) of nuclei suspension were mixed with 200 μl of 1% low melting point agarose (Sigma-Aldrich) at 37°C and gently transferred onto slides. The gel was covered with a cover glass, slides were cooled on ice for 1 min. Cover glasses were removed and slides were immersed in lysing solution (2.5 M NaCl, 100 mM EDTA, 10 mM Tris HCl pH 7.5) for 20 min at room temperature. After lysis, slides were washed twice in the neutral solution TBE (89 mM Tris Base, 89 mM Boric Acid, 2 mM EDTA, pH 8.3) for 5 min, rinsed in 70% ethanol (v/v) for 10 min at room temperature. Slides were stained with 20 μl DAPI (4′-6-diamidine-2′-phenylindole dihydrochloride; Sigma-Aldrich, 1 μg/ml). One hundred nuclei were analyzed per slide. Cells undergoing PCD or necrosis were distinguished from viable cells as indicated by Singh (2003). In case of PCD, cell nuclei showed an undefined outline without any clear boundary due to nucleosomal-sized DNA diffusing into the agarose. Necrotic cell nuclei were bigger and poorly defined.

### Quantitative Real-Time Polymerase Chain Reaction (RT-qPCR)

RNA isolation was carried out using the Aurum^TM^ Total RNA Fatty and Fibrous Tissue Kit (Bio-Rad, Milan, Italy), according to manufacturer’s instructions. cDNAs were obtained with the High Capacity cDNA Reverse Transcription Kit (Applied Biosystems, Monza, Italy) according to supplier’s suggestions. Gene-specific oligonucleotide primers for the *M. truncatula MtTdp2α* (Phytozome Database Accession No. Medtr8g146980) and *MtTop2* (Phytozome Database Accession No. Medtr3g103270), coding sequences were designed using the Real-Time PCR Primer Design program from GenScript^1^ (Supplementary Table S1). RT-qPCR reactions were carried out on cDNAs using the Maxima^®^ SYBR Green qPCR Master Mix (Thermo Fisher Scientific, Milan, Italy) in a final volume of 10 μl according to supplier’s indications, and using a Rotor-Gene 6000 PCR apparatus (Corbett Robotics, Brisbane, QLD, Australia). Amplification conditions included an initial denaturation step at 95°C for 10 min, and subsequently 95°C for 30 s, 59°C for 60 s, 72°C for 30 s (40 cycles). For each oligonucleotide set, a non-template control (water) was also included. The *M. truncatula ELF1α* (GenBank Accession No. EST317575) was used as a reference gene, based on previous work (Donà et al., 2013) (Supplementary Table S1). Three independent samples for each experimental condition (transgenic versus non-transgenic, etoposide-treated, and untreated cells) were amplified in technical triplicates. The raw, background-subtracted, fluorescence data provided by the Rotor-Gene 6000 Series Software 1.7 (Corbett Robotics) was used to estimate PCR efficiency (E) and threshold cycle number (Ct) for each transcript quantification. The Pfaffl method (Pfaffl et al., 2002) was used for relative quantification of the studied transcript accumulation and statistic analysis using the REST2009 Software V2.0.13 (Qiagen GmbH, Hilden, Germany).

### Statistical Analysis

For each treatment, two independent experiments were performed, using three replications. Asterisks indicate statistically significant differences determined using Student’s *t*-test (^∗^*P* < 0.10, ^∗∗^*P* < 0.05, ^∗∗∗^*P* < 0.01).

## Results

### *MtTdp2α* Gene Overexpression Features Expansion of Nucleolar Area and Occurrence of Nucleolar Vacuoles

**FIGURE 1:**
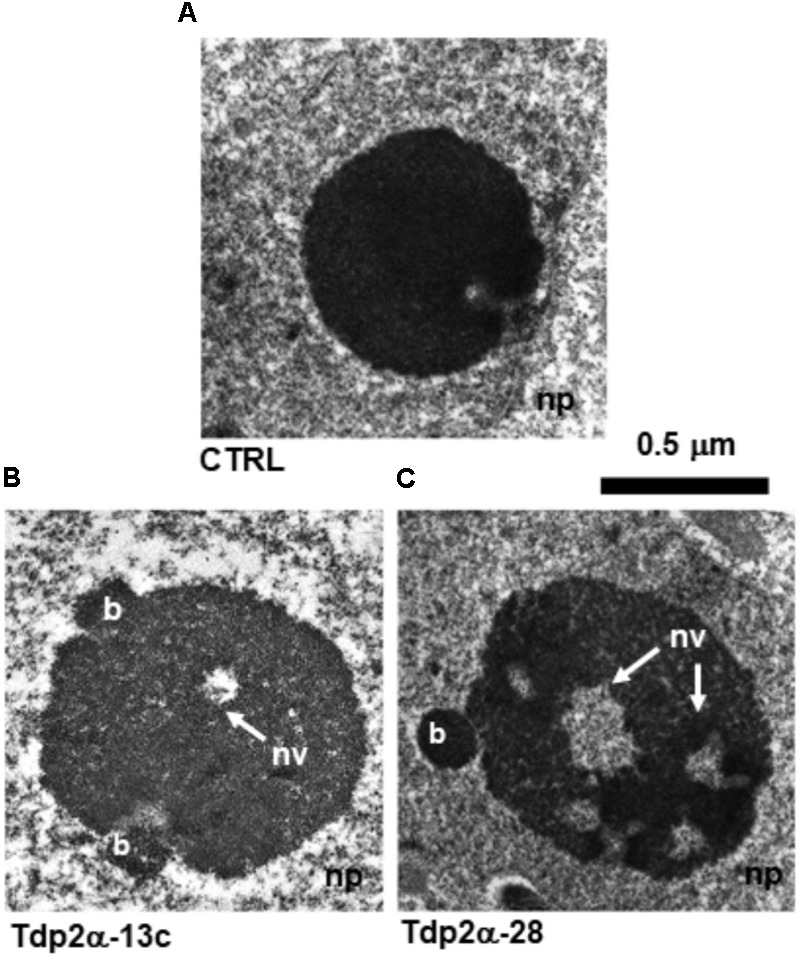
Ultrastructural analysis reveals that overexpression of *MtTdp2α* gene associates with increased nucleolar area and occurrence of nucleolar vacuoles. Four-day old proliferating *Medicago truncatula* suspension cultures were invstigated: **(A)** control (CTRL) cells characterized by a compact, roundish nucleolus. Increased nucleolar size was observed in the *MtTdp2α*-overexpressing lines Tdp2α-13c and Tdp2α-28 **(B,C)**. The occurrence of peripheral budding structures (b) and nucleolar vacuoles (nv, arrows) is evidenced. np, nucleoplasm.

The nucleolus ultrastructure was revealed by transmission electron microscopy (TEM) carried out on 4-day-old *M. truncatula* suspension cultures grown under physiological conditions. The proliferative state of *M. truncatula* cells was assessed by monitoring the expression profiles of the *MtH4* (histone H4) gene (Supplementary Figure S1). CTRL nucleoli were round and compact (**Figure 1A**), while the *MtTdp2α*-overexpressing cells showed expanded and decondensed nucleoli (**Figures 1B,C**). Budding fibrillar structures (b) were observed at the nucleolar periphery in Tdp2α-13c and Tdp2α-28 lines (**Figures 1B,C**). The appearance of such small bodies has been reported as an early sign of nucleolar decondensation (Stepinski, 2014). The estimated average area of the CTRL nucleoli was 0.63 ± 0.15 μm^2^. A significant increase was observed in *MtTdp2α*-overexpressing cells with an average nucleolar area of 1.04 ± 0.18 μm^2^ (Tdp2α-13c) and 0.91 ± 0.29 μm^2^ (Tdp2α-28), corresponding to 34 and 44% expansion of the nucleolar region, respectively (**Table 1**). In the nucleoli of *MtTdp2α*-overexpressing cells, TEM highlighted the presence of cavities (also known as nucleolar vacuoles) (**Figures 1B,C**; nv, arrows), another peculiarity of actively transcribing nucleoli (Stepinski, 2014). The number of vacuoles ranged from a few small ones to a single, visibly larger, cavity located in the central region of the nucleolus. The occurrence of nucleolar vacuoles in CTRL and *MtTdp2α*-overexpressing cells was quantified, resulting in an average number of 0.5 ± 0.3 (CTRL), 2.3 ± 0.91 (Tdp2α-13c), and 3.2 ± 1.4 (Tdp2α-28) cavities per nucleolus (**Table 1**). Taken together, the reported data highlight changes in the nucleolus morphology and ultrastructure of *MtTdp2α*-overexpressing cells, compatible with conditions of enhanced nucleolar activity.

**Table 1:** Morphometric analysis of nucleoli.

Line	Nucleolar area (μm^2^)	Number of nucleolar vacuoles
CTRL	0.63 ± 0.19	0.5 ± 0.3
Tdp1α-13c	1.04 ± 0.17^∗∗∗^	2.3 ± 0.91^∗∗∗^
Tdp1α-28	0.91 ± 0.29^∗∗∗^	3.2 ± 1.4^∗∗∗^

*^∗∗∗^*P* < 0.001.*

### Occurrence of Multinucleolate Cells and Ring-Shaped Nucleoli in *MtTdp2α-*Overexpressing Lines

**FIGURE 2:**
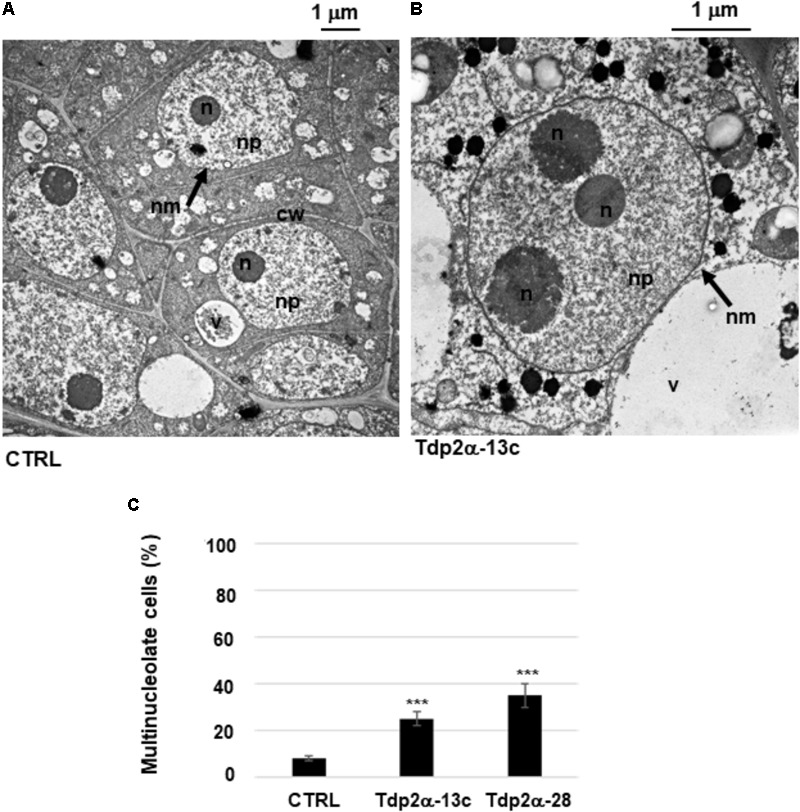
Occurrence of multinucleolate cells in *MtTdp2α*-overexpressing lines. *M. truncatula* cells with a single nucleolus (CTRL line) **(A)** and a multinucleolate cell of the *MtTdp2α*-overexpressing line Tdp2α-13c **(B)** revealed by transmission electron microscopy (TEM). n, nucleolus; nm, nuclear membrane; np, nucleoplasm; cw, cell wall; v, vacuole. **(C)** Morphometric analysis evidenced the distribution of multinucleolate cells in 4-day old suspension cultures of CTRL, Tdp2α-13c, and Tdp2α-28 lines. Data represent the mean ± SD of three replicates per experiment. Asterisks indicate statistically significant differences determined using Student’s *t*-test (*P* < 0.05).

Morphometric analysis showed that the percentage of multinucleolate cells was significantly higher in the *MtTdp2α-*overexpressing lines compared to CTRL. Examples of mononucleolate and multinucleolate cells are shown in **Figures 2A,B**. The multinucleolate cells accounted for 8.3 ± 1.1% of the total population in the control suspension culture (**Figure 2C**, CTRL) while a significant increase (25.0 ± 3.2%, 3.0-fold) in the percentage of cells containing more than one nucleolus (up to three nucleoli in most cases) was observed in the Tdp2α-13c line, compared to CTRL (**Figure 2C**). The percentage of multinucleolate cells was further enhanced in the Tdp2α-28 line, with an estimated value of 35.0 ± 4.8% (4.37-fold) compared to CTRL (**Figure 2C**).

**FIGURE 3:**
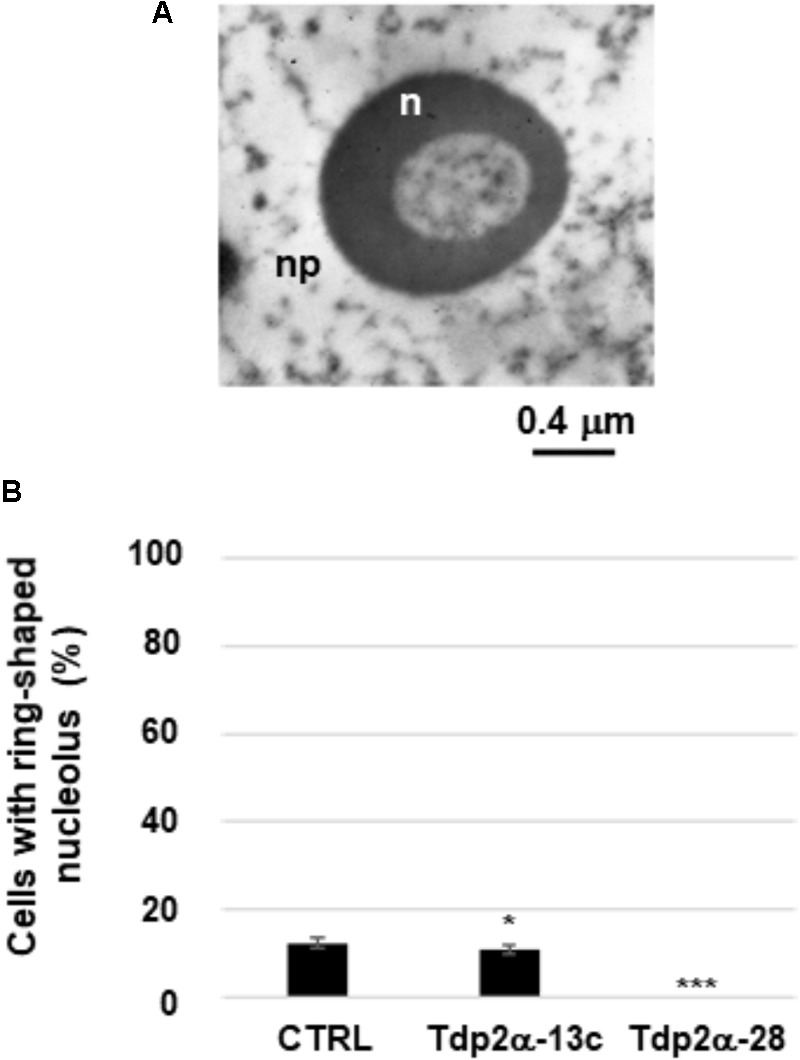
Occurrence of ring-shaped nucleoli in *MtTdp2α*-overexpressing lines. **(A)** Ultrastructural morphology of a ring-shaped nucleolus (Tdp2α-13c line). n, nucleolus; np, nucleoplasm. **(B)** Distribution of ring-shaped nucleoli in 4-day old suspension cultures of CTRL, Tdp2α-13c, and Tdp2α-28 lines. Data represent the mean ± SD of three replicates per experiment. Asterisks indicate statistically significant differences determined using Student’s *t*-test (*P* < 0.05).

In the *M. truncatula* cell suspensions collected at 4 days of culture, TEM revealed the occurrence of ring-shaped nucleoli characterized by a central vacuolization surrounded by a nucleolar shell (**Figure 3A**). According to the current literature, ring-shaped nucleoli correspond to a reversible quiescent state generally induced in plant cells by stress conditions (Balcerzak et al., 2011). *MtTdp2α* gene overexpression correlated with a reduction in the number of ring-shaped nucleoli. Quantitative analysis showed that 12.5 ± 1.2% of CTRL cells contained ring-shaped nucleoli. The percentage of cells containing ring-shaped nucleoli was significantly reduced in the Tdp2α-13c line (11.0 ± 0.9%) whereas no ring-shaped nucleoli were present in the Tdp2α-28 line (**Figure 3B**).

### Increased Transcriptional Activity in Nucleolar and Extra-Nucleolar Regions of *MtTdp2α*-Overexpressing Cells

**FIGURE 4:**
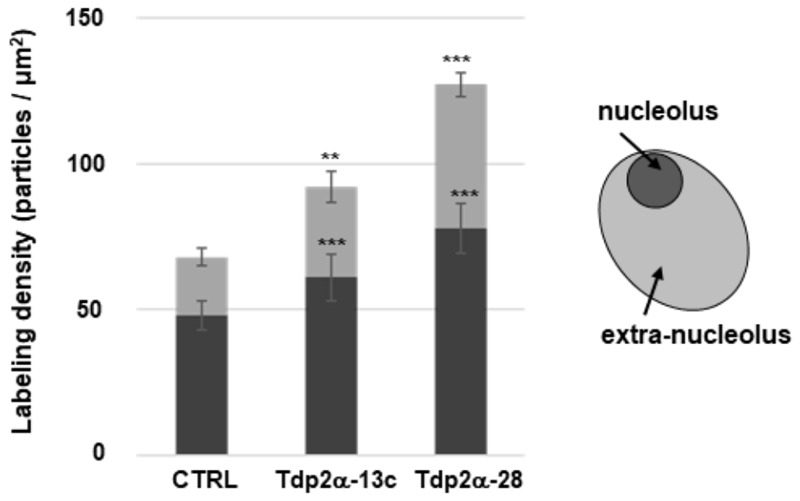
Increased transcriptional activity is observed in the nucleolar and extra-nucleolar regions of *MtTdp2α*-overexpressing cells. Quantitative evaluation of anti-BrU immunolabeling. BrU (100 mM) was provided for 1 h to 4-day old *M. truncatula* cell suspension cultures (CTRL and *MtTdp2α*-overexpressing lines) and BrU incorporation into transcripts was measured in the nucleolar (dark gray) and extra-nucleolar (pale gray) regions. A schematic representation of the nucleus with highlighted nucleolar and extra-nucleolar areas is shown. np, nucleoplasm. The frequency of labeled transcripts is expressed as number of gold particles per μm^2^. Data represent the mean ± SD of three replicates per experiment. Asterisks indicate statistically significant differences determined using Student’s *t*-test (*P* < 0.05).

Quantitative evaluation of BrU incorporation was performed in both nucleolar and extra-nucleolar regions (**Figure 4**). As for CTRL, the estimated transcript number in the nucleolus was 48.00 ± 5.07 particles per μm^2^. A significant increase was detected in Tdp2α-13c and Tdp2α-28 lines (61.00 ± 7.98 and 78.00 ± 8.51 particles per μm^2^, respectively) (**Figure 4**, dark gray). The quantitative evaluation in the region outside the nucleolus revealed as well an enhanced distribution of labeled transcripts in the *MtTdp2α*-overexpressing lines, compared to CTRL. The estimated values were 20.00 ± 3.10 particles per μm^2^ (CTRL), 31.00 ± 5.45 particles per μm^2^ (Tdp2α-13c), and 49.00 ± 3.97 particles per μm^2^ (Tdp2α-28) (**Figure 4**, pale gray). Thus, *MtTdp2α* gene overexpression was associated with increased transcription levels. The overall transcriptional response within the nucleus corresponded to 68.00 ± 8.00 particles per μm^2^ (CTRL), 92.00 ± 13.4 particles per μm^2^ (Tdp2α-13c), and 127.00 ± 11.5 particles per μm^2^ (Tdp2α-28). The enhancement in transcription reported for the *MtTdp2α*-overexpressing cells well-correlates with the results from ultrastructural morphometric analysis, providing further evidence of increased nucleolar activity.

To assess the possible correlation between *MtTdp2α*gene overexpression and rRNA processing, *q*RT-PCR-based analysis of the 5.8S rRNA unspliced precursor and mature forms was carried out in *M. truncatula* cells treated with MG-132, a selective inhibitor of 26S proteasome (see Supplementary Data, Supplementary Figure S2). The reported data demonstrate that overexpression of *MtTdp2α* gene was associated with enhanced ribogenesis rate and increased tolerance to the proteasome inhibitor MG-132.

### Overexpression of *MtTdp2α* Gene Confers Tolerance to Etoposide

**FIGURE 5:**
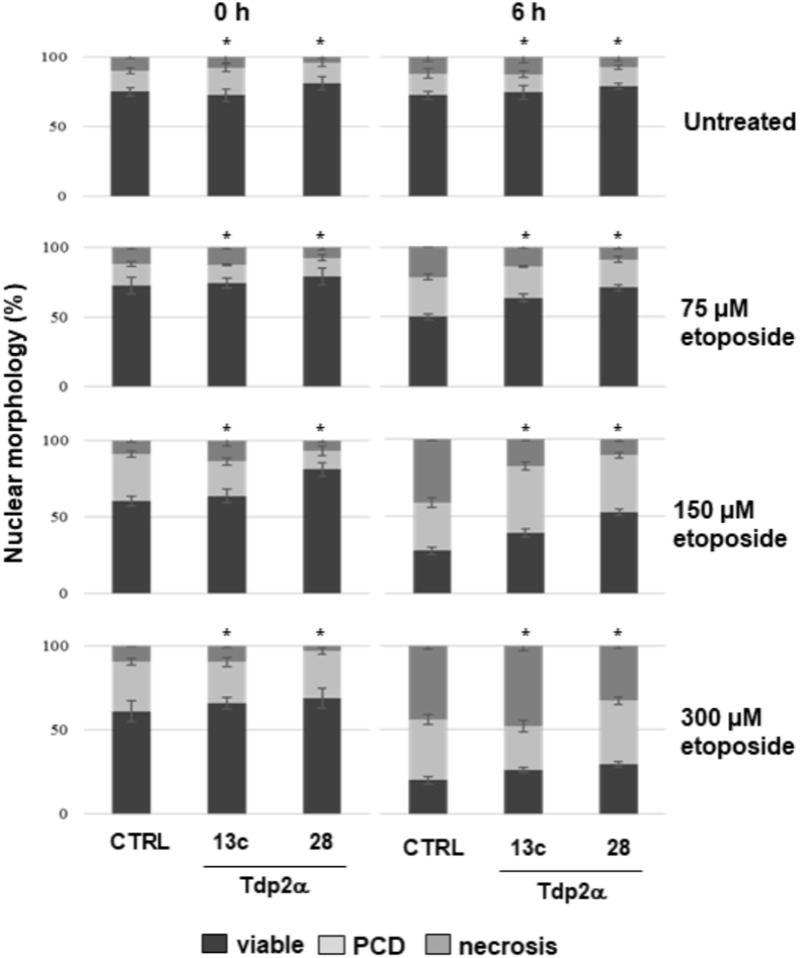
Overexpression of *MtTdp2α* gene confers tolerance to etoposide. *MtTdp2α*-overexpressing lines Tdp2α-13c and Tdp2α-28 and CTRL line exposed to increasing etoposide concentrations (0, 75, 150, and 300 μM) were analyzed using the DNA diffusion test at 0 and 6 h following treatments. The percentage of viable, PCD and necrotic cells is shown. For each treatment combination, data represent the mean values of three independent replications. Asterisks indicate statistically significant differences determined using Student’s *t*-test (*P* < 0.05).

Four-day-old *M. truncatula* suspension cultures were incubated with increasing concentrations (0, 75, 150, and 300 μM) of the topo II inhibitor etoposide. The dose range was chosen based on the previous study by Locato et al. (2006). Cell death was monitored at 0 and 6 h following treatments, using the DNA diffusion assay. Three distinct nuclear morphologies, corresponding to viable cells, PCD and necrosis events were evidenced. In the untreated samples, a significant higher percentage of viable cells (81.0 ± 4.5%) was recorded in the Tdp2α-28 line, compared to Tdp2α-13c (72.5 ± 4.5%) and CTRL (75.0 ± 3.0%) lines (**Figure 5**). Following exposure to 75 μM etoposide, the CTRL showed only 50.0 ± 2.2% viable cells at 6 h whereas viability was significantly higher in both Tdp2α-13c and Tdp2α-28 lines (63.6 ± 5.5 and 71.0 ± 2.0% viable cells, respectively). When 150 μM etoposide was provided, cytotoxic effects were evident at 0 h in the CTRL line, possibly due to the time required for sample processing. At 6 h, a drop in the percentage of viable cells (27.6 ± 2.2%) was observed in the CTRL line. PCD and necrosis frequencies accounted for 31.0 ± 3.0 and 41.4 ± 1.2%, respectively (**Figure 5**). The *MtTdp2α*-overexpressing lines showed a significantly higher the percentage of viable cells (39.1 ± 2.5%, Tdp2α-13c; 52.6 ± 2.1%, Tdp2α-28). PCD events occurred with frequency of 43.5 ± 2.5% (Tdp2α-13c) and 36.8 ± 2.1% (Tdp2α-28), while necrosis was observed at lower frequency (17.4 ± 1.1% Tdp2α-13c; 10.5 ± 1.5% Tdp2α-28). The highest dose (300 μM) resulted into 20.0 ± 2.2% viability in CTRL, with PCD and necrosis accounting for 36.0 ± 3.1 and 44.0 ± 2.9%, respectively (**Figure 5**).

It has been reported that the level of imposed stress is crucial for determining the fate of plant cells and that the PCD/necrosis ratio is useful in understanding the stress response of cell populations (Reape and McCabe, 2008). The PCD/necrosis ratio was calculated for all the tested lines and etoposide concentrations at 6 h, since at this timepoint following stress exposure the first PCD events can be detected (Balestrazzi et al., 2010). In the CTRL line, the ratio was reduced (from 1.29 to 0.75–0.81) when the 150 and 300 μM etoposide doses were provided, clearly indicating the dose-dependent predominance of necrotic cell death events (Supplementary Table S2). In both *MtTdp2α*-overexpressing lines, there was a progressive increase in the PCD/necrosis ratio with the 75 and 150 μM etoposide doses (Supplementary Table S2). This finding shows that, differently from CTRL, the Tdp2α-13c and Tdp2α-28 cells perceived the 75 and 150 μM inhibitor concentrations as a mild stress, allowing them to activate PCD. The observed ability of *MtTdp2α*-overexpressing lines to withstand etoposide-mediated cytotoxicity provides evidence of the possible link between Tdp2 and Topo II functions in plant cells.

### Expression of *MtTdp2α* and *MtTop2* Genes Under Etoposide Treatment

**FIGURE 6:**
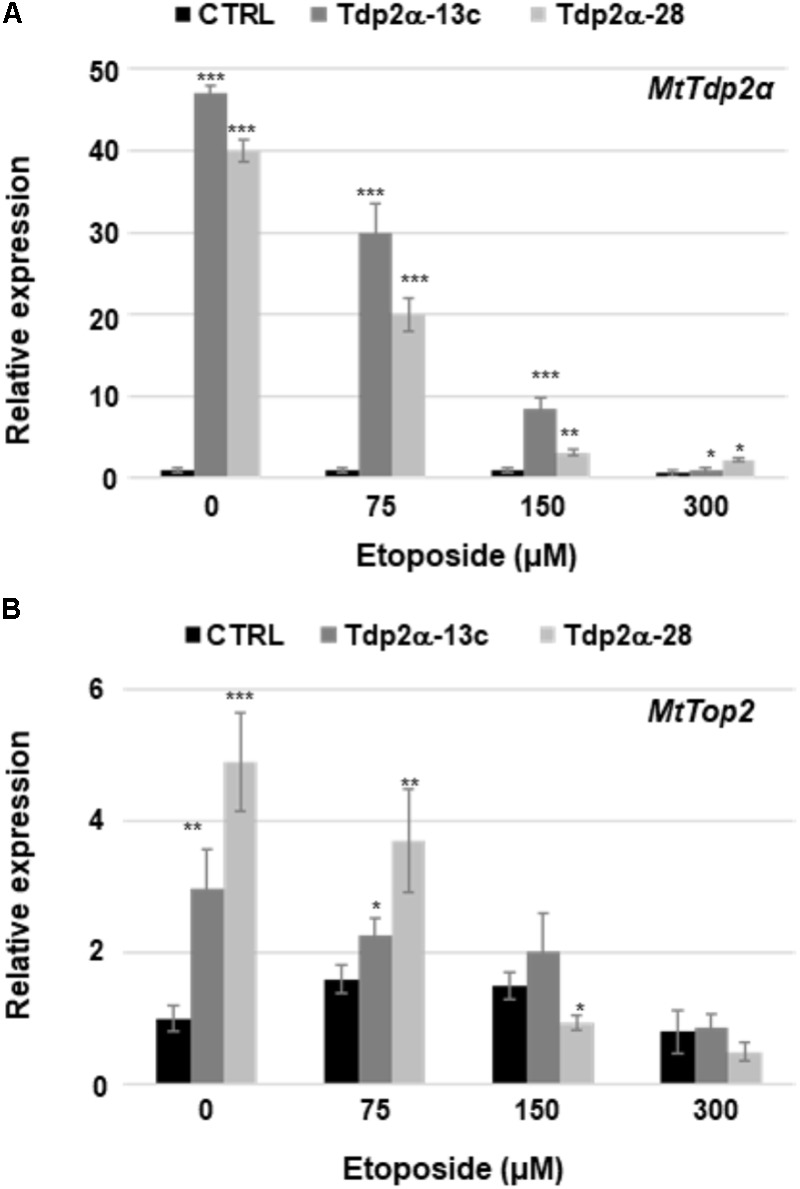
*MtTdp2α* gene overexpression was associated with the ability to withstand etoposide-mediated cytotoxicity. Transcript levels of the *MtTdp2α* **(A)** and *MtTop2* **(B)** genes in *M. truncatula* cell suspension cultures (Tdp2α-13c, Tdp2α-28, and CTRL line) exposed to etoposide (0, 75, 150, and 300 μM). RT-qPCR analysis was carried out at 6 h following treatments. For each treatment combination, data represent the mean values of three independent replications. Asterisks indicate statistically significant differences determined using Student’s *t*-test (*P* < 0.05).

Etoposide exerts its cytotoxic effects by stabilizing the transient topo II-DNA covalent complexes which result in increased levels of protein–DNA adducts subsequently processed into DSBs. In animal cells, Tdp2 is able to resolve the covalent link between topo II and DNA by directly excising these adducts (Cortes Ledesma et al., 2009). In the absence of etoposide, the level of *MtTdp2α* mRNA was 47- and 40-fold higher in the Tdp2α-13c and Tdp2α-28 lines, respectively, compared to CTRL (**Figure 6A**). In response to 75 μM etoposide, the level of *MtTdp2α* transcript was still significantly higher (30-fold, Tdp2α-13c; 20-fold, Tdp2α-28) at 6 h of treatment with the inhibitor. At the same timepoint, the *MtTdp2α* mRNA amount further decreased in cells treated with 150 μM etoposide, however the transcript levels remained significantly higher (8.4-fold, Tdp2α-13c; 3.1-fold, Tdp2α-28) compared to CTRL. Finally, in response to 300 μM etoposide, there was a drop in the expression level of *MtTdp2α* gene, in agreement with the low frequency of viable cells previously reported for this treatment (**Figure 6A**). *MtTdp2α*-overexpressing cells showed significant up-regulation of the *MtTop2* gene, encoding topo II, under physiological conditions (**Figure 6B**), as previously described (Araujo et al., 2016). In the absence of inhibitor, the amount of *MtTop2* transcript was 2.0- and 4.0-fold higher in Tdp2α-13c and Tdp2α-28 cells compared to CTRL. In response to 75 μM etoposide, the level of *MtTop2* transcript was still significantly higher (0.5-fold, Tdp2α-13c; 1.5-fold, Tdp2α-28) at 6 h of treatment with the inhibitor, compared to CTRL. At the same timepoint, the *MtTop2* mRNA amount did not significantly change in Tdp2α-13c cells treated with 150 μM etoposide whereas the transcript levels were decreased in Tdp2α-28, compared to CTRL. Finally, in response to 300 μM etoposide, there was a drop in the expression level of *MtTop2* gene, in agreement with the cytotoxic effects of the treatment (**Figure 6B**). The observed overexpression of *MtTop2* gene might contribute to etoposide tolerance in Tdp2α-13c and Tdp2α-28 lines.

### Effects of Etoposide on Nucleolar Morphology

**FIGURE 7:**
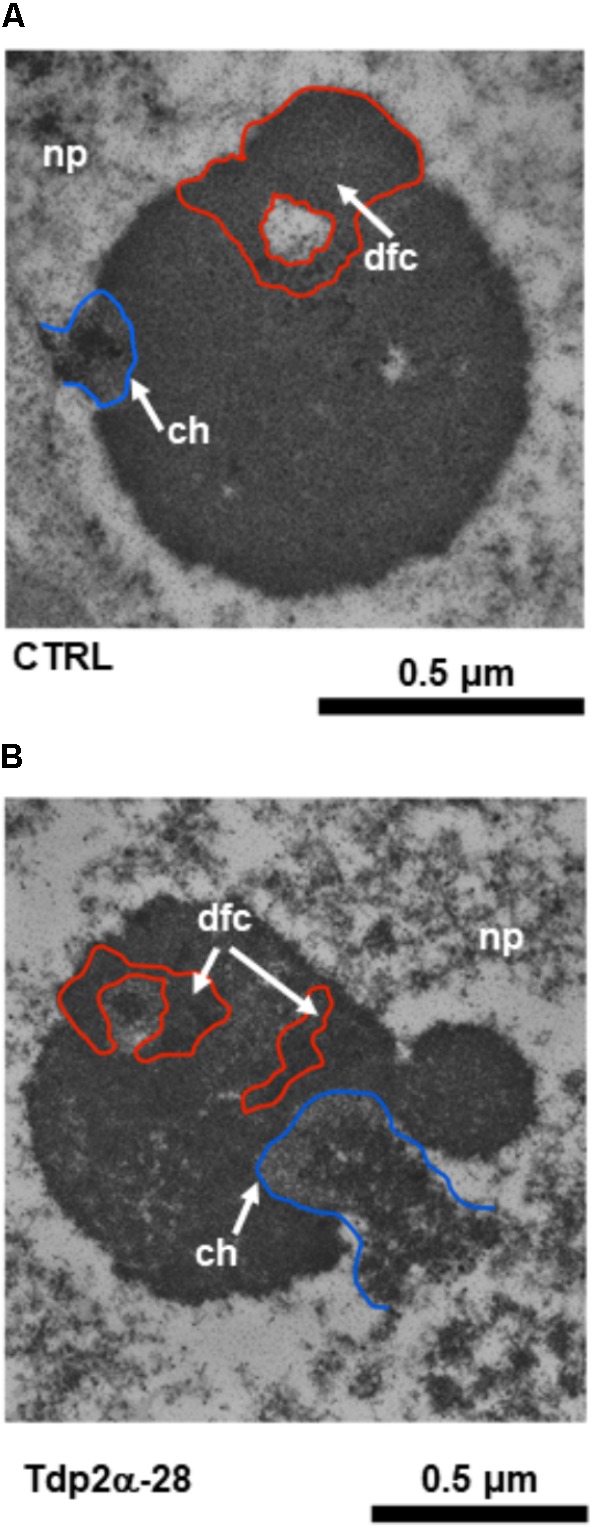
Rearrangements of nucleolar morphology in *MtTdp2α*-overexpressing lines exposed to etoposide. Ultrastructural morphology of the nucleolus in *M. truncatula* cells (**A**, CTRL and **B**, Tdp2α-28 lines) challenged with 150 μM etoposide. The blue line surrounds the chromatin (ch, arrow) areas from which the nucleolus is stemming out. The red line surrounds the Dense Fibrillar Component (dfc, arrow).

Based on results of DNA diffusion assay, the treatment with 150 μM etoposide was then selected for ultrastructural analysis. In both CTRL and *MtTdp2α*-overexpressing cells, exposure to etoposide resulted in morphological features resembling the so-called nucleolar caps which in animal cells have been associated with the response to transcriptional stress and DNA repair (Shav-Tal et al., 2005; van Sluis and McStay, 2015). One of these morphological hallmarks, located at the nucleolar periphery, is evident in the CTRL nucleolus shown in **Figure 7A** (arrow). Other distinct morphological features were evident in the etoposide-treated nucleoli of *M. truncatula* cells and the corresponding nucleolar regions have been highlighted in **Figure 7** using a colored line (**Figures 7A,B**, red and blue lines). The blue line surrounds the chromatin (ch) areas from which the nucleolus is stemming out. As shown in **Figures 7A,B** (blue line), the region involved in the release of nucleolar material into the surrounding nucleoplasm is visibly larger in the Tdp2α-28 nucleolus, compared to CTRL. This was a distinctive feature of *MtTdp2α*-overexpressing cells (data not shown). Another morphological change is evidenced by the red line which surrounds the dense fibrillar component (dfc) or the site of rRNA processing (**Figures 7A,B**, red line and arrow). Differences in the fragmentation and distribution of DFC areas were observed between CTRL and *MtTdp2α*-overexpressing cells. The finding that these rearrangements were more frequently detected in the *MtTdp2α*-overexpressing lines would further support the idea that transcription is maintained and enhanced under stress conditions in the Tdp2α-13c and Tdp2α-28 lines.

## Discussion

In the present work, several ultrastructural morphological hallmarks were identified in the *M. truncatula* lines overexpressing the *MtTdp2α* gene: expansion of the nucleolar area, occurrence of ring-shaped nucleoli, presence of nucleolar vacuoles, increased nucleolar activity, and resistance to cytotoxicity.

As for the increased nucleolar area, found in active nucleoli of highly proliferating cells (Bassy et al., 2000), many reports have so far evidenced the positive correlation between nucleolus size and activity. In rapidly dividing cancer cells, the amount of proteins involved in ribosome biogenesis is enhanced, causing nucleolar hypertrophy (Derenzini et al., 1998; Pena et al., 2001; Takada and Kurisaki, 2015). *M. truncatula* lines overexpressing the *MtTdp2α* gene also showed an increased number of nucleoli per cell. To date, information concerning the mechanisms which determine the number of nucleoli per cell is still scanty (McCann and Baserga, 2014). In a recent work (Chen et al., 2016), the occurrence of enlarged nucleoli and multinucleolate cells has been associated with the overexpression of the *THAL (THALLO)* gene in *Arabidopsis*. The THAL protein, member of the conserved SAS10 (SOMETHING ABOUT SILENCING 10)/C1D family with functions in pre-rRNA processing, has a peculiar role in the modulation of the nucleolar function, contributing to both rDNA transcription and pre-rRNA processing. According to these authors, hypertrophic nucleoli and multinucleolate cells might result from ectopic rDNA transcription and dispersal (Chen et al., 2016). In other cases, nucleolar hypertrophy has been reported in *Arabidopsis* mutants showing defects in ribosome biogenesis and impaired cell cycle (Steinborn et al., 2002; Tzafrir et al., 2002; Lahmy et al., 2004). Evidence of the proliferative state of *MtTdp2α*-overexpressing cells has been shown by Araujo et al. (2016) and further confirmed by the expression profiles of the *MtH4* gene, hereby reported. Based on this, the observed ultrastructural features might reflect the enhanced nucleolar activity of the proliferating *MtTdp2α*-overexpressing cultures. In agreement with this explanation, TEM revealed that *MtTdp2α* gene overexpression correlated with a reduction in the number of ring-shaped nucleoli. In both animal and plant cells, the occurrence of ring-shaped nucleoli has been so far recognized as a stress indicator. In blood cells, ring-shaped nucleoli correspond to a reversible quiescent state associated with a reduction in the rRNA transcription (Smetana, 2002). Exposure to lead increased the frequency of ring-shaped nucleoli in *Lupinus* root cells, compared to the untreated control (Balcerzak et al., 2011). A similar finding was reported in starved cells of *Zea mays* (Kuran and Kononowicz, 1979) and in *Glycine max* cells exposed to chilling (Stepinski, 2010). It has been also hypothesized that the ring-shaped morphology might derive from enhanced RNA synthesis and transport outside the nucleolus (Stepinski, 2010; Balcerzak et al., 2011). The observed decrease in the number of ring-shaped nucleoli suggests for the improved ability of *MtTdp2α*-overexpressing cells to withstand stress, in agreement with previous reports showing the increased expression of DDR and antioxidant genes in the Tdp2α-13c and Tdp2α-28 lines in absence/presence of stress agents (Confalonieri et al., 2014; Faè et al., 2014; Araujo et al., 2016). Furthermore, *MtTdp2α*-overexpressing plants could buffer DSBs accumulation under both physiological and stress conditions (Confalonieri et al., 2014; Faè et al., 2014).

Results from TEM analysis raise the discussion on the occurrence and function of nucleolar vacuoles in *MtTdp2α*-overexpressing cells. According to Shaw and Brown (2004), the nucleolar vacuole is a highly dynamic entity, devoid of transcription, whose presence and structure is dependent upon cell cycle progression. As reviewed by Stepinski (2014), nucleolar vacuoles are formed in the most active nucleoli when ribonucleoprotein particles are transferred from the nucleolus to the cytoplasm at rates higher than the rate of synthesis. It is possible that the presence of nucleolar vacuoles in *MtTdp2α*-overexpressing cells might correlate with enhanced nucleolar activity.

Ultrastructural analyses provided novel insights into the nucleolar architecture of plant cells overexpressing the *MtTdp2α* gene, strengthening the positive correlation between *MtTdp2α* overexpression and nucleolus performance. The expansion in nucleolar size detected in *MtTdp2α*-overexpressing cells also correlated with BrU-incorporation profiles. The latter revealed a significant increase in transcription not only within the nucleolar area but also in the extra-nucleolar region. It is possible that the observed enhancement in nucleolar transcription occurring in the *MtTdp2α*-overexpressing cells might be due to pre-rRNA overaccumulation resulting from higher rates of rDNA transcription. Vilotti et al. (2011) demonstrated that participation of human Tdp2 in rRNA processing requires the SIM (SUMO-Interacting) motif while the 5′-tyrosyl DNA phosphodiesterase activity is dispensable. Since the SIM motif is absent from the plant Tdp2α protein, it could be hypothesized that the same role might be played by the zinc finger RanBP2 domain, found in the plant protein (Faè et al., 2014). The RanBP2 domain is part of a novel class of RNA binding domain able to bind single-stranded RNA (Nguyen et al., 2011) and involved in chloroplast RNA editing (Sun et al., 2015). Future in-depth studies will be necessary to elucidate this specific aspect of the plant Tdp2 function.

DNA damage response is a highly balanced system. Selection toward more effective systems for genome maintenance should be expected. On the other hand, adaptation essential for surviving results from uncorrected mutations. These two opposing forces interacting each other, thus influencing influence the overall structure and activity of the DDR network (Aravind et al., 1999). In animal cells, TDPs are deeply embedded in the DDR network with their documented ability to interact with DDR players specifically involved in different repair pathways. Thus, there is evidence for a novel role of TDPs in the cross-talk between the repair pathways. Moreover, in animal cells TDP2 participates in signaling pathways that control proliferation/differentiation/apoptosis. It is reasonable to hypothesize that such multipurpose enzymes need to be kept under strict control at different levels, including the transcriptional level.

*MtTdp2α* gene overexpression was associated with the ability to withstand etoposide-mediated cytotoxicity. This is in agreement with the first observation made by Cortes Ledesma et al. (2009) who noted that *Tdp2* gene overexpression significantly increased etoposide resistance in yeast. Interestingly, both the *MtTdp2α*-overexpressing lines showed a significant reduction in the percentage of necrosis events when exposed to the drug. Similarly, a reduction in the number of necrotic cells was recorded in the Tdp2α-13c and Tdp2α-28 plants grown *in vitro* in the presence of osmotic stress (Confalonieri et al., 2014) and heavy metal stress (Faè et al., 2014). The necrosis frequency increases when a cell population is exposed to high-stress levels which cause physical injury, leading to cell disruption (Reape and McCabe, 2008). It is reasonable to hypothesize that both the 75 and 150 μM etoposide doses, which resulted in high necrosis frequency in the CTRL line, were perceived by the *MtTdp2α*-overexpressing lines as a mild stress with limited impact in terms of necrosis. On the other hand, considering the well-characterized role played by the human Tdp2 as a signal transduction component, the involvement of *MtTdp2α* gene in intracellular signaling pathways controlling PCD in plants cannot be excluded. Two main features of the *MtTdp2α*-overexpressing lines contributed to etoposide tolerance. First, significantly higher levels of the *MtTdp2α* transcript were maintained in the Tdp2α-13c and Tdp2α-28 cells exposed to increased drug concentrations and, second, the up-regulation of *MtTop2* gene. Topo II plays an essential role in the complex process that allows DNA package into chromatids, acting in concert with cohesin to generate DNA loops (Razin et al., 1991; Fajkus et al., 1998; Nasmyth, 2017; Shintomi et al., 2017). Upon exposure to anticancer drugs, such as etoposide, topo II becomes a persistent source of DSBs. Whether up-regulation of *MtTop2* and several other DDR genes, previously reported for the *MtTdp2α*-overexpressing cells (Faè et al., 2014; Araujo et al., 2016), represents a general response of the cell machinery to the perturbation of *MtTdp2α* gene expression or instead is the consequence of enhanced signaling mediated by the *MtTdp2α* gene itself, remains an open question. A puzzling aspect of this study is the finding that Tdp2α-13c cells always showed significantly higher levels of *MtTdp2α* transcript in presence/absence of etoposide whereas *MtTop2* gene expression followed an opposite trend. Such ‘non-correlated fluctuations’ between the *MtTdp2α* gene overexpression in the expression of other genes were also reported in previous studies carried at the plant (Confalonieri et al., 2014; Faè et al., 2014) and cell suspension level (Araujo et al., 2016). At the moment, it is difficult to figure whether the observed differences between Tdp2α-13c and Tdp2α-28 lines, in terms of secondary effects, might be related to genome positional effects or gene activation by transgene insertion. It is possible that the overexpression of MtTdp2α gene in Tdp2α-13c line overpasses a critical threshold and that beyond this threshold no further protein is produced or some cellular pathways are influenced (Prelich, 1999). Overall, it should be underlined that there is an evident causality relationship between the two *MtTdp2α*-overexpressing lines showing the same trend while differing in the intensity of the response at the phenotype level.

In animal cells, the Tdp1 enzyme also contributes to the repair of topo II-mediated DNA damage (Murai et al., 2012). As for *M. truncatula* cells, only slight up-regulation of *MtTdp1α* gene was observed in Tdp2α-13c and Tdp2α-28 lines treated with etoposide (Macovei, personal communication). Treatments with the drug caused changes in the nucleolar morphology, highlighting the presence of structures resembling the so-called nucleolar caps (Shav-Tal et al., 2005; van Sluis and McStay, 2017). Structures similar to those hereby observed in *M. truncatula* cells exposed to etoposide have been described by Shav-Tal et al. (2005) in HeLa cells treated with actinomycin D. These authors refer to small and convex shaped nucleolar caps attached to the nucleolus central body that arise under stress conditions. TEM analysis suggested that nucleolar caps derive from changes in the nucleolar structure reflecting the segregation of the granular and fibrillar components. According to Shav-Tal et al. (2005), the nucleolus responds to impairment of transcription by redistributing its components to better cope with stress until physiological levels of transcription will be resumed. Several studies have highlighted the involvement of nucleolar caps in the DDR. van Sluis and McStay (2015) demonstrated that accumulation of DSBs within the rDNA triggers the ATM-dependent transcriptional block followed by nucleolar reorganization. This rearrangement brings the damaged rDNA region within caps at the nucleolar surface where several DDR proteins are recruited (van Sluis and McStay, 2015). Nucleolar cap-like structures were observed in *Arabidopsis* cells upon treatments with phosphorylation and transcription inhibitors (Tillemans et al., 2006). Since the ultrastructural hallmarks resembling caps and other nucleolar rearrangements were particularly frequent in etoposide-treated *MtTdp2α*-overexpressing cells, this might be an additional evidence of the response orchestrated by an extremely active nucleolus against cytotoxic and genotoxic stress.

Although the *MtTdp2α* expression level was higher in Tdp2α-13c cells than Tdp2α-28, the observed phenotypic effects were always stronger in the Tdp2α-28 line. In this context, it should be considered the possibility that, as reported in animal cells, also in plant cells the *Tdp2* gene might play a dual role, in the DNA repair processes as well as in the proliferation-related signaling pathways. At the moment, we cannot rule out any involvement of the *MtTdp2α* gene in plant signaling networks. This will be starting point for future investigations that will provide global profiles and additional information concerning the *MtTdp2α* gene function.

## Conclusion

To date, many aspects of the plant response to genotoxic stress are still poorly investigated, despite their relevance in crop stress response within the scenario of global climate change. A useful way to investigate mechanisms involved in the plant response to genotoxic stress, and possibly disclose novel interactions, relies on the availability of cell and/or plant lines having altered expression of DDR genes as demonstrated in the present work for the Tdp2 function. This novel player in the stress response mediated by the plant nucleolus might be a useful tool to unravel the stress sensing mechanisms that act within the ‘nucleolar surveillance systems’ in plants.

## Author Contributions

AB, AM, and SdSA conceived the work and wrote the manuscript. AM, MF, and MB contributed to the experimental data. MB performed TEM analyses. AB, AM, SdSA, MB, and DC contributed to the discussion of data. All authors read and agreed with the final version of the manuscript.

## Conflict of Interest Statement

The authors declare that the research was conducted in the absence of any commercial or financial relationships that could be construed as a potential conflict of interest. The reviewer TT and handling Editor declared their shared affiliation.

## Footnotes

1 https://www.genscript.com/ssl-bin/app/primer
