# Supplementary material for: Ultrastructural and Molecular Analyses Reveal Enhanced Nucleolar Activity in Medicago truncatula Cells Overexpressing the MtTdp2α Gene
Source: Front Plant Sci. 2018 May 11;9:596. doi: 10.3389/fpls.2018.00596 (PMC5958304; doi:10.3389/fpls.2018.00596)
Supplement: Supplementary file 1 [file Data_Sheet_1.DOCX]

**Supplemental Data**

**Ultrastructural and molecular analyses reveal enhanced nucleolar activity in *Medicago truncatula* cells overexpressing the *MtTdp2α* gene**

Anca Macovei, Matteo Faè, Marco Biggiogera, Susana Araujo, Daniela Carbonera, Alma Balestrazzi

**Expression profiles of proliferation marker gene *MtH4***

The expression profiles of *MtH4* (*HISTONE H4*) gene, regarded as S-phase associated indicator, were monitored in order to assess the proliferation state of CTRL and *MtTdp2α*-overexpressing *M. truncatula* cells collected at four days of culture. As shown in Supplemental Fig. S1, a significant increase in *MtH4* transcript levels was observed in four-day old Tdp2α-13c (P1 = 0.03 > 0.05) and Tdp2α-28 (P2 = 0.01 > 0.05) suspension cultures, compared to CTRL.

**Materials and Methods**

Total RNA was isolated using the Aurum Total RNA Fatty and Fibrous Tissue kit (Bio-Rad, Milan, Italy). One microgram of RNA was reverse-transcribed using the iScript cDNA Synthesis kit (Bio-Rad) and the poly-T oligonucleotide primer. Gene-specific oligonucleotide primers for the *MtH4* gene (Medtr7g114370.1) (H4-Forward: 5’-CCGTAAGGTGCTTCGTGATA-3’; H4-Reverse: 5’-CAAACCGCTTATACGCTTCA-3’) were designed using the RealTime PCR Primer Design program from GenScript (https://www.genscript.com/ssl-bin/app/primer). QRT-PCR was carried out with the SsoFast™ EvaGreen® Supermix (Bio-Rad), according to supplier’s indications, in a Rotor-Gene 6000 Real Time PCR apparatus (Corbett Robotics, Brisbane, Australia). Amplification conditions were as follows: initial denaturation step at 95 °C for 30 s, and subsequently 95 °C for 5 s, 59 °C for 30 s, 72 °C for 30 s (40 cycles). For each oligonucleotide set, a non-template control (water) was included. The *M. truncatula ELF1α* (GenBank #EST317575) (Supplemental Table S1) was used reference gene (Doná et al. 2013; Confalonieri et al. 2014; Faè et al. 2014). The PfaffI method (Pfaffl 2002) was used for relative quantification of transcript accumulation. Statistic analysis was performed with REST2009 Software V2.0.13 (Qiagen GmbH, Hilden, Germany). Results were subjected to analysis of variance (ANOVA). Percentage data were transformed to arcsin√x before statistical analysis. Statistical significance of differences was determined using Student’s *t*-test (**P* < 0.05).

**References**

**Confalonieri M, Faè M, Balestrazzi A, Donà M, Macovei A, Valassi A, Giraffa G, Carbonera D.** 2013. Enhanced osmotic stress tolerance in *Medicago truncatula* plants overexpressing the DNA repair gene *MtTdp2α* (tyrosyl-DNA phosphodiesterase 2). Plant Cell Tissue and Organ Culture **116,** 187-203.

**Donà M, Ventura L, Balestrazzi A, Buttafava A, Carbonera D, Confalonieri M, Giraffa G, Macovei A.** 2013b. Dose-dependent reactive species accumulation and preferential double strand breaks repair are featured in the γ-ray response in *Medicago truncatula* cells. Plant Molecular Biology Reporter **32,** 129-141.

**Faè M, Balestrazzi A, Confalonieri M, Donà M, Macovei A, Valassi A, Giraffa G, Carbonera D.** 2014. Copper-mediated genotoxic stress is attenuated by the overexpression of the DNA repair gene *MtTdp2α* (tyrosyl-DNA phosphodiesterase 2) in *Medicago truncatula* plants. Plant Cell Reports **33,** 1071-1080.

**Pfaffl MW, Horgan GW, Dempfle L.** 2002. Relative expression software tool (REST) for group-wise comparison and statistical analysis of relative expression results in real-time PCR. Nucleic Acid Research **30,** e36.

**Supplemental Fig. S1.** Transcript levels of *MtH4 (HISTONE H4)* gene evaluated by *q*RT-PCR in proliferating (four-day old) *M. truncatula* cell suspension cultures of control (CTRL), MtTdp2α-13c and MtTdp2α-28 lines grown under physiological conditions. Data represent the mean values ± SD of three independent replications. Asterisks indicate statistically significant differences determined using Student’s *t*-test (‘*’ *P* < 0.10, ‘**’ *P* < 0.05, ‘***’ *P* < 0.01).

***MtTdp2α* gene overexpression associates with enhanced rRNA transcription and tolerance to the proteasome inhibitor MG-132**

To assess the possible correlation between *MtTdp2α *gene overexpression and rRNA processing, *q*RT-PCR-based analysis of the 5.8S rRNA unspliced precursor and mature forms was carried out in *M. truncatula* cells. The schematic representation of the rDNA region is shown in Supplemental Fig. S2(A). Four-day old *M. truncatula* cells were exposed for 4 h to 50 μM MG-132, a selective inhibitor of 26S proteasome.

The 5.8S rRNA precursor which contains the Internal Transcribed Spacer (ITS) 1 and the mature 5.8S rRNA, released following excision of the ITS1 region, are shown in Supplemental Fig. S2(B and C, respectively). Treatment with MG-132 significantly reduced (40%) the level of 5.8S rRNA unspliced precursor in CTRL line, compared to the untreated sample. By contrast, no significant changes in the amount of 5.8S rRNA unspliced precursor were observed in the Tdp2α-13c and Tdp2α-28 cells exposed to MG-132, compared to untreated cells of the same lines. Following MG-132 exposure, the amount of 5.8S rRNA precursor significantly increased (up to 1.0-fold and 0.6-fold) in the Tdp2α-13c and Tdp2α-28 lines, respectively, compared to CTRL line (Supplemental Fig. S2, B).

As for the 5.8S rRNA mature form, the CTRL line treated with the proteasome inhibitor showed an estimated reduction of 25% while both Tdp2α-13c and Tdp2α-28 lines revealed enhanced accumulation.The amount of 5.8S mature rRNA significantly increased (up to 0.8-fold and 1.5-fold) in Tdp2α-13c and Tdp2α-28 lines, respectively, compared to the CTRL line (Supplemental Fig. S2, B). Furthermore, the estimated amount of 5.8S rRNA mature was slightly but significantly increased (0.2-fold, Tdp2α-13c; 0.8-fold, Tdp2α-28), respectively, compared to the untreatedcells of the same lines. The reported data demonstrate that overexpression of *MtTdp2α* gene was associated with enhanced ribogenesis rate and increased tolerance to the proteasome inhibitor MG-132.

**Materials and Methods**

RNA isolation, cDNA synthesis and *q*RT-PCR were carried as previously described. Oligonucleotide primers 5.8S-F (5’-TCCCGTGAACCATCGAGTCT-3’) and 5.8S-R (5’- CCCTCAACCTAATGGCATCG-3’) were designed to amplify the full 5.8S rRNA sequence (mature ribosomal RNA, GenBank accession AF233339.1). Oligonucleotide primers ITS1-F (5’-ACGGTTTTCGTGCGTGTTGT-3’) and 5.8S-R were used to amplify the region spanning from the 5’-end of the Internal Transcribed Spacer 1 (ITS1) to the 3’-end of 5.8S RNA. ITS1 is spliced out from the precursor transcript to generate the mature 5.8S rRNA.

**Supplemental Fig. S2.** (**A**) Schematic representation of the rDNA region. (**B**) Representation of the 5.8S rRNA precursor which contains the ITS (Internal Transcriber Spacer) 1 and mature 5.8S rRNA. The latter is released following excision of the ITS1 region. Levels of precursor 5.8S rRNA measured by *q*RT-PCR in four-day old *M. truncatula* cell suspension cultures (CTRL line and *MtTdp2α*-overexpressing lines Tdp2α-13c and Tdp2α-28) incubated for 4 h in absence (CTRL) or presence of MG-132. (**C**) The 5.8S rRNA mature form. Levels of mature 5.8S rRNA measured by *q*RT-PCR in four-day old *M. truncatula* cell suspension cultures (CTRL line and *MtTdp2α*-overexpressing lines Tdp2α-13c and Tdp2α-28) incubated for 4 h in absence (CTRL) or presence of MG-132.

Arrows indicate the annealing sites of the oligonucleotide primers used for *q*RT-PCR analysis. 1, ITS1-F primer. 2, 5.8S-F primer. 3, 5.8S-R primer. Asterisks indicate statistically significant differences determined using Student’s *t*-test (‘*’ *P* < 0.10, ‘**’ *P* < 0.05, ‘***’ *P* < 0.01).

**Supplemental Table S1.** Sequences of oligonucleotide primers utilized in *q*RT-PCR.

| **Gene** | **Forward Primer** | **Reverse Primer** | **Efficiency** |
| --- | --- | --- | --- |
| *MtTdp2α* | 5'-CAGATGTTCAGCAAGGAACG-3' | 5'-CCCGTCTTGCAAAGGATATT-3' | 1.78 |
| *MtTop2* | 5'-AGGATCCGTGGGATTGTAAGGC-3' | 5'-ACAACAGAGAGGCCAGCCATAG-3' | 1.91 |
| *MtELF1* | 5'-GACAAGCGTGTGATCGAGAGATT-3' | 5'-TTTCACGCTCAGCCTTAAGCT-3' | 1.90 |

**Supplemental Table S2.** PCD/necrosis ratio calculated for CTRL and MtTdp2α-overexpressing lines at 6 h following exposure to increasing etoposide concentrations

| **Line Etoposide (μM)**    **0 75 150 300** |
| --- |
| CTRL 1.00 1.29 0.75 0.81  Tdp2α-13c 1.04 1.58 2.50 0.50  Tdp2α-28 1.80 2.22 3.50 1.25 |
